# Mechanical thrombectomy of distal cerebral vessel occlusions of the anterior circulation

**DOI:** 10.1038/s41598-023-32634-0

**Published:** 2023-04-07

**Authors:** Dominik Sepp, Moritz Roman Hernandez Petzsche, Teresa Zarth, Silke Wunderlich, Benno Ikenberg, Christian Maegerlein, Claus Zimmer, Maria Teresa Berndt, Tobias Boeckh-Behrens, Jan Stefan Kirschke

**Affiliations:** 1 Department of Diagnostic and Interventional Neuroradiology, Klinikum rechts der Isar, School of Medicine, Technical University of Munich, München, Germany; 2 Department of Neurology, Klinikum rechts der Isar, School of Medicine, Technical University of Munich, Ismaninger Straße 22, 81675 München, Germany

**Keywords:** Stroke, Neurological disorders

## Abstract

Mechanical thrombectomy (MT) is frequently performed for distal medium vessel occlusions (DMVO) of the anterior circulation in acute stroke patients. However, evidence for its clinical benefit remains scarce. In this study, we aim to investigate clinical course and safety outcomes of MT in comparison to standard medical therapy (SMT) in DMVO. This single-center retrospective observational study included 138 consecutive patients treated for DMVO of the anterior circulation between 2015 and 2021. To reduce the risk of selection bias, propensity score matching (PSM) of patients with MT versus SMT was performed for the covariates NIHSS and mRS at admission. Out of all 138 patients, 48 (34.8%) received MT and 90 (65.2%) received SMT only. Overall, patients treated with MT showed significantly higher NIHSS and mRS scores at admission. Post 1:1 PSM, there was a trend toward a better NIHSS improvement in patients with MT (median 4 vs. 1, *P* = 0.1). No significant differences were observed in the occurrence of symptomatic intracranial hemorrhage or mortality between the groups before and after PSM. A subgroup analysis showed significantly higher NIHSS improvement (median 5 versus 1, *P* = 0.01) for patients with successful MT (≥ mTICI 2b). Mechanical thrombectomy for distal medium vessel occlusions (DMVO) in the anterior circulation appeared safe and feasible. Successful recanalization was associated with clinical improvement. Larger, multi-center, randomized-controlled trials are required to corroborate these findings.

## Introduction

Over the last decade, several randomized-controlled trials (RCTs) have demonstrated the clinical efficacy of mechanical thrombectomy (MT) in the treatment of large vessel occlusion (LVO) of the anterior circulation in acute stroke patients^1–5^. These paradigm studies have established MT as a cornerstone of LVO treatment. The inclusion criteria of these studies were almost exclusively limited to patients with occlusions in the carotid-T, the M1 segment or the proximal M2 segment of the middle cerebral artery.

Improvement of stent-retriever devices and endovascular techniques now regularly enables successful reperfusion of distal medium vessel occlusions (DMVOs) of the anterior circulation located in the distal M2 and the M3 segment^6,7^ as well as the ACA^2,8^. Although DMVOs are clinically more variable and often manifest in milder symptoms than LVOs, affected patients frequently suffer from severe disability. Therefore, endovascular treatment of DMVOs in the anterior circulation is regularly attempted in comprehensive stroke centers.

However, experiences gained for LVOs in the above mentioned RCTs cannot be unquestionably transferred to DMVOs, as those have a more variable clinical presentation, better response to intravenous thrombolysis (IVT)^9^ and higher complexity of the endovascular procedure due to smaller vessel size as well as higher vessel tortuosity^10^. The benefit of endovascular therapy in the treatment of DMVOs in the anterior circulation remains to be conclusively evaluated to establish clear medical guidelines for the use of MT in DMVOs.

This retrospective single-center case–control study aims to investigate the clinical benefits and safety outcomes of mechanical thrombectomy in acute stroke patients with DMVO in the anterior circulation and to compare them with the outcomes of standard medical treatment (SMT) in daily clinical practice.

## Methods

### Study population

This retrospective single-center case control study included consecutive patients who were admitted between January 1st, 2015 and March 31st, 2021 for ischemic stroke at our comprehensive stroke center based on the following criteria: 1. Diagnosis of an acute ischemic stroke due to an occlusion of the anterior cerebral artery (ACA) in the A1–A3 segment or 2. Diagnosis of an acute ischemic stroke due to an occlusion of the middle cerebral artery in the distal (defined as the latter half) M2 segment or the M3 segment. Tandem lesions with occlusion of the ipsilateral ICA were excluded. The diagnosis was made using a standard CT stroke protocol including CT head, arterial CT angiography, and perfusion CT. In some cases, primary stroke MRI was performed including fluid attenuated inversion recovery (FLAIR), diffusion weighted imaging (DWI), and time of flight (TOF) angiography sequences.

The localization of the occlusion was verified by two experienced neuroradiologists (DS and JSK). All patients received either mechanical thrombectomy (MT) or standard medical therapy (SMT), including stroke unit care with or without intra-venous thrombolysis (IVT) according to standard medical guidelines. MT was performed using approved aspiration catheters and stent-retrievers. Sex, age, National Institutes of Health Stroke Scale (NIHSS) score, modified Rankin Scale (mRS), clinical history, and therapeutic data were acquired from the medical records. The stroke etiology was classified according to the Trial of Org 10,172 in Acute Stroke Treatment (TOAST) criteria^11^. After interventional therapy, reperfusion was quantified based on the modified Thrombolysis in Cerebral Infarction (mTICI) scale^12^ by two independent neuroradiologists.

### Clinical outcome

The clinical parameters mRS and NIHSS were assessed at admission and at discharge by neurologists. NIHSS and mRS improvement was calculated by subtracting the score at discharge from the score at admission, higher scores therefore indicate a higher NIHSS or mRS improvement. Symptomatic intracranial hemorrhage was classified in accordance with the Second European-Australasian Acute Stroke (ECASS II) Study^13^. Mortality rates for the duration of the in-hospital stay was recorded.

### Statistical analysis

Normally distributed metric data was described as mean and standard deviation (SD). Metric data lacking normal distribution as well as ordinal data were described as median and interquartile range (IQR). Categorical data was described using absolute and relative frequencies.

1:1 propensity score matching (PSM) was performed to diminish selection bias. The propensity score was estimated using a logistic regression model adjusted for NIHSS and mRS scores on admission using the SPSS PSM algorithm with a caliper of 0.15.

Patients who underwent mechanical thrombectomy (MT) were statistically compared to patients who received standart medical therapy (SMT). Normally distributed metric variables were compared using an unpaired, two-tailed t test. Non-normally distributed metric and ordinal variables were compared using the Mann–Whitney-U test. Categorical variables were compared using the χ^2^ Test or the Fisher’s exact test if distributed on a 2 × 2 crosstab. *P* values ≤ 0.05 were considered statistically significant. Statistics were performed using SPSS 26 (IBM Corporation).

### Ethical approval

This study was approved by the local ethics board (“Ethikkommission der Technischen Universität München”) in accordance with local and regional law and has therefore been performed in accordance with the ethical standards laid down in the 1964 Declaration of Helsinki and its later amendments. The requirement of patient informed consent was waived by the ethics committee due to the retrospective nature of the study in accordance with local law.

## Results

### Baseline parameters

Between January 2015 and March 2021, 138 Patients were treated for acute occlusions in the distal part of the M2 Segment, the M3 segment, and the ACA. Average patient age was 76 years. The most common cardiovascular risk factor was hypertension (60.1%), followed by atrial fibrillation (50.7%). Out of all patients, 48 (34.8%) underwent mechanical thrombectomy (MT).

Due to significant differences in the admission parameters NIHSS and mRS between MT and SMT groups (median 10.5 vs. 5 and 4 vs. 3, all *P* < 0.001; Table 1), PSM was performed for these parameters. A total of 82 patients remained after PSM without NIHSS or mRS baseline differences (*P* > 0.2).

**Table 1:** Baseline parameters before and after PSM.

	Before PSM				After 1:1 PSM			
	All Patients	MT	SMT	P	All Patients	MT	SMT	P
N	138	48	90		82	41	41	
%		34.8%	65.2%			50.0%	50.0%	
*Age*								
Mean	76.4	76.9	76.2	0.8	76.8	75.7	77.9	0.4
Male sex (N)	64	24	40	0.5	41	20	21	1.0
%	46.4%	50.0%	44.4%		50.0%	48.8%	51.2%	
A. fibrillation (N)	70	31	39	**0.02**	49	26	23	0.6
%	50.7%	64.6%	43.3%		59.8%	63.4%	56.1%	
Hypertension (N)	83	32	51	0.3	50	26	24	0.8
%	60.1%	66.7%	56.7%		61.0%	63.4%	58.5%	
Diabetes (N)	31	15	16	0.1	17	11	6	0.28
%	22.5%	31.3%	17.8%		20.7%	26.8%	14.6%	
Hyperlipidemia (N)	24	10	14	0.5	15	7	8	1.0
%	17.4%	20.8%	15.6%		18.3%	17.1%	19.5%	
*Pre-Stroke mRS*								
Median	0	0	0	**0.001**	0	0	0	0.09
IQR	0.0–0.0	0.0–1.25	0.0–0.0		0.0–0.0	0.0–1.0	0.0–0.0	
Missing data (N)	29	10	19		19	9	10	
*Admission mRS*								
Median	3	4	3	**0.000**	4	4	4	0.23
IQR	2.0–4.0	3.0–5.0	1.0–4.0		3.0–5.0	3.0–5.0	3.0–4.0	
*Admission NIHSS*								
Median	7	10.5	5	**0.000**	9.5	10	8	0.25
IQR	3.0–12.0	7.0–15.0	2.0–8.50		5.0–13.25	6.0–14.5	4.0–12.0	
*Occlusion site*								
MCA (N)	106	32	74	0.11	57	28	29	0.97
	76.8%	66.7%	82.2%		69.5%	68.3%	70.7%	
ACA (N)	25	12	13		21	11	10	
	18.1%	25.0%	14.4%		25.6%	26.8%	24.4%	
Both (N)	7	4	3		4	2	2	
	5.1%	8.3%	3.3%		4.9%	4.9%	4.9%	
*TOAST*								
Macroangiopathy (N)	9	1	8	**0.010**	4	1	3	0.53
%	6.6%	2.1%	8.9%		4.9%	2.4%	7.3%	
Cardio-embolic (N)	77	34	43		53	29	24	
%	55.8%	70.8%	47.8%		64.6%	70.7%	58.5%	
Other (N)	4	3	1		3	2	1	
%	2.9%	6.3%	1.1%		3.7%	4.9%	2.4%	
Unknown (N)	48	10	38		22	9	13	
%	34.8%	20.8%	42.2%		26.8%	22.0%	31.7%	
*Recanalization degree*								
Below TICI 2b		13 (27.1%)				11 (26.8%)		
TICI 2b or better		35 (72.9%)				30 (73.2%)		
IV Thrombolysis (N)%	52	19	33	0.9	30	17	13	0.49
	37.7%	39.6%	36.7%		36.6%	41.5%	31.7%	

Significant values are shown in bold.

*SMT* standard medical therapy, *MT* mechanical thrombectomy, *IQR* interquartile range, *mRS* modified rankin scale, *NIHSS* national institute of health stroke scale, *TOAST* trial of org 10,172 in acute stroke treatment, *TICI* thrombolysis in cerebral infarction score, *PSM* propensity score matching.

Vascular occlusions were found predominantly in the MCA territory in 106 (76.8%) patients, in the ACA territory in 25 (18.1%) patients, and in both territories in 7 (5.1%) patients. There was no significant difference in the occlusion localization between the groups. The distribution of vascular occlusions remained similar before and after PSM. The most common cause of stroke as classified by the TOAST criteria was cardio-embolism (55.8% of the study cohort). No underlying stroke etiology was found in 34.8% of the study cohort. Intravenous thrombolysis (IVT) was administered in a total of 52 (37.7%) cases, there were no significant differences in the rates of administered IVT between the groups (*P* = 0.9). After PSM the rate of IVT remained similar.

**Figure 1:**
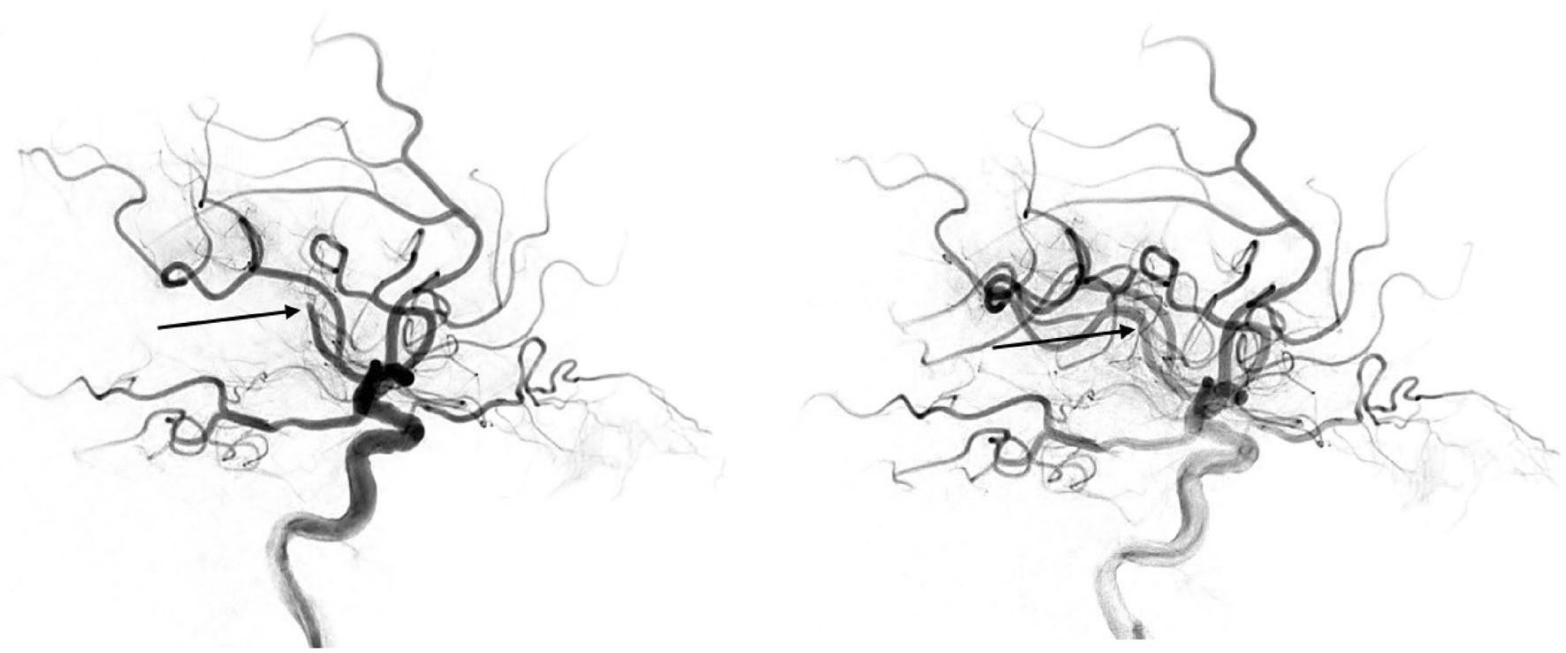
MT for a distal M2 occlusion. Pre-interventional angiographic finding (left) showing the arterial occlusion (arrow) at the M2/M3 junction of the inferior branch of the inferior trunk of the middle cerebral artery. After stent-retriever assisted MT, successful revascularization of the occlusion (mTICI 2c, right). NIHSS at admission: 19, NIHSS at discharge: 7.

Successful revascularization of at least mTICI 2b was achieved in 35 (72.9%) patients who underwent mechanical thrombectomy. After PSM, successful revascularization was achieved in 30 (73.2%) patients. Figure 1 shows an exemplary case of successful MT in a patient with a distal M2 occlusion.

### Clinical and safety outcome

In the complete cohort without PSM, both mRS disability score and NIHSS score at discharge were significantly higher in MT patients than in the SMT group, reflecting significantly higher mRS and NIHSS scores at admission (*P* < 0.001, Table 2).

**Table 2:** Outcome parameters before and after PSM.

	Before PSM				After 1:1 PSM			
	All	MT	SMT	P	All	MT	SMT	P
*NIHSS at discharge*								
Median	2.0	7.0	2.0	**0.001**	5.0	5.0	4.0	0.77
IQR	1.0–9.0	2.0–13.0	0.25–5.75		1.0–10.5	1.0–10.0	1.0–12.0	
*NIHSS improvement*								
Median	2.0	4.0	1.5	**0.033**	2.5	4.0	1.0	0.10
IQR	0.0–5.0	0.0–6.75	0.0–4.0		0.0–6.0	0.0–6.5	0.0–5.0	
*mRS at discharge*								
Median	2.0	4.0	2.0	**0.001**	3.0	3.0	3.0	0.71
IQR	1.0–4.0	1.0–5.0	1.0–3.0		1.0–4.75	1.0–5.0	1.0–4.0	
*mRS improvement*								
Median	0.0	1.0	0.0	0.290	0.0	1.0	0.0	0.38
IQR	0.0–1.0	− 0.75–2.0	0.0–1.0		0.0–2.0	− 1.0–2.0	0.0–1.0	
mRS improvement ≥ 1 (N)	63	26	37	0.14	37	22	15	0.12
%	45.7%	54.2%	41.1%		45.1%	53.7%	36.6%	
*Safety*								
SH (N)	4	2	2	0.610	2	1	1	1.0
%	2.9%	4.2%	2.2%		2.4%	2.4%	2.4%	
iHM (N)	11	5	6	0.514	9	4	5	1.0
%	8.0%	10.4%	6.7%		11.0%	9.8%	12.2%	

Significant values are shown in bold.

*SH* symptomatic intracranial hemorrhage, *iHM* in-house Mortality, *SMT* standard medical therapy, *MT* mechanical thrombectomy, *IQR* interquartile range, *mRS* modified rankin scale, *NIHSS* national institute of health stroke scale, *PSM* propensity score matching.

On the other hand, NIHSS improvement was significantly higher in the MT group before PSM (median 4.0 vs. 1.5, *P* < 0.05). After PSM, there was no significant difference in mRS or NIHSS at discharge (*P* > 0.7) with a non-significant trend toward a higher NIHSS improvement in the MT group (median 4.0 vs. 1.0, *P* = 0.1).

Median mRS improvement was slightly higher in the MT group both before and after PSM without achieving statistical significance. Similarly, a non-significantly higher percentage of patients experienced a mRS improvement of at least 1 point (before PSM: 54.2% in the MT group vs. 41.1% in the SMT group, *P* > 0.1 and after PSM: 53.7% in the MT group vs. 36.6% in the SMT group, *P* > 0.1).

The patient cohorts were evaluated for the occurrence of symptomatic intracranial hemorrhage (SH) as suggested in the Second European-Australasian Acute Stroke (ECASS II) Study^13^. In the complete cohort, SH occurred in four patients (2.9%). No significant differences were found between the two cohorts regarding this safety end-point before and after PSM. Additionally, occurrence of in-house mortality (iHM) was evaluated as the second relevant safety end-point. A total of 11 patients (8.0%) died in the immediate post-stroke hospital stay. As with SH, no significant frequency differences were found regarding iHM between the two cohorts (*P* > 0.5, Table 2).

Periprocedural complications occurred in five of 48 MT patients (10.4%). Four patients (8.3%) developed groin bleedings, with one requiring interventional treatment. Two patients (4.2%) had an embolization in new territories (ENT) without clinical deterioration.

### Subgroup analysis

**Figure 2:**
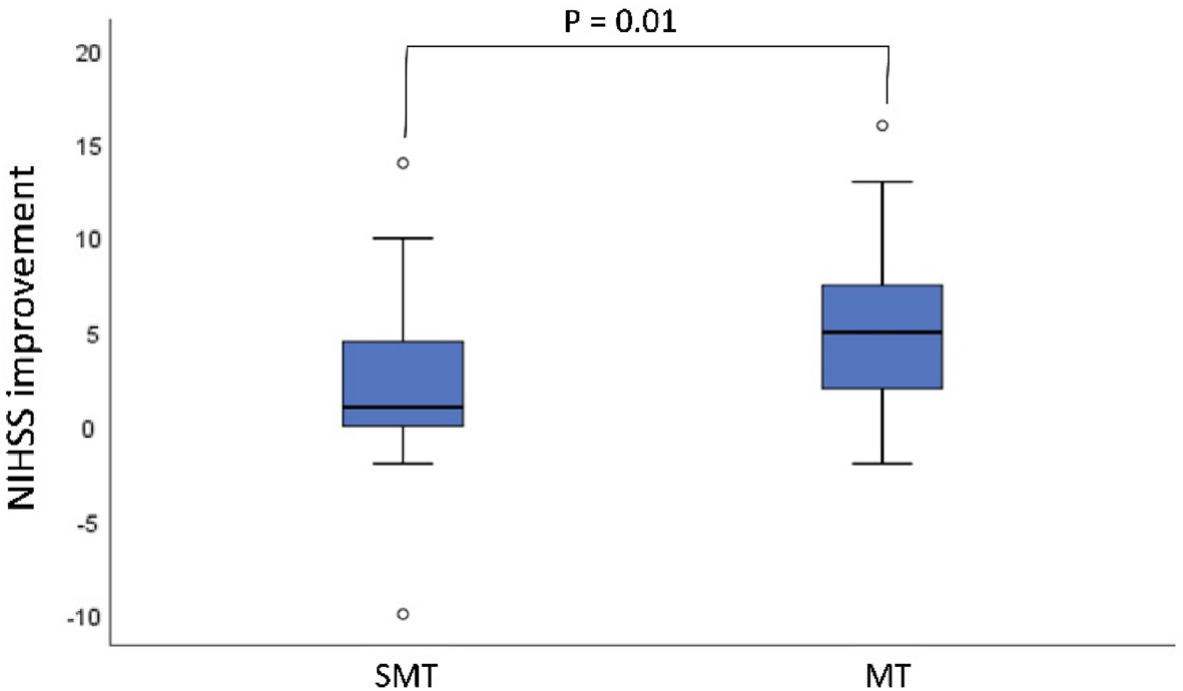
Box-plot of NIHSS improvement between admission and discharge after PSM.

In a subgroup analysis, patients who underwent successful MT, defined as a reperfusion result of mTICI 2b or better, were compared to the SMT group (Table 3a). Both before and after PSM, patients with successful MT showed a higher mRS improvement (median 1.0 vs. 0.0, *P* < 0.1) and a significantly higher NIHSS improvement (after PSM: median 5.0 vs. 1.0, *P* = 0.01) than patients who received SMT (Fig. 2). Similarly, a significantly higher percentage of patients experienced a mRS improvement of at least one point (after PSM 63.3% vs. 36.6%, *P* < 0.05). No significant difference was found in the frequency of symptomatic intracranial hemorrhage or in-house mortality.

**Table 3:** Subgroup comparison of patients with successful recanalization (≥ TICI 2b) with the SMT cohort (Table 3a) and patients with unsuccessful recanalization (< TICI 2b) with the SMT cohort (Table 3b).

**3a**						
	Before PSM			After 1:1 PSM		
	MT	SMT	P	MT	SMT	P
Subgroup	≥ TICI 2b			≥ TICI 2b		
N	35	90		30	41	
*NIHSS improvement*						
Median	5	1.5	**0.000**	5	1.0	**0.010**
IQR	1.0–8.0	0.0–4.0		1.0–8.25	0.0–5.0	
*mRS improvement*						
Median	1	0	**0.024**	1	0.0	0.098
IQR	0.0–2.0	0.0–1.0		0.0–2.25	0.0–1.0	
mRS improvement ≥ 1 (N)	23	37	**0.017**	19	15	**0.027**
%	65.7%	41.1%		63.3%	36.6%	
SH (N)	1	2	1.0	1	1	1.0
%	2.9%	2.2%		3.3%	2.4%	
iHM (N)	3	6	0.709	3	5	1.0
%	8.6%	6.7%		10.0%	12.2%	

**3b**						
	Before PSM			After 1:1 PSM		
	MT	SMT	P	MT	SMT	P
Subgroup	< TICI 2b			< TICI 2b		
N	13	90		11	41	
*NIHSS improvement*						
Median	0	1.5	0.115	0	1	0.304
IQR	− 5.0–2.0	0.0–4.0		− 5.0–2.0	0.0–5.0	
*mRS improvement*						
Median	0	0	0.083	0	0	0.274
IQR	− 1.0–1.0	0.0–1.0		− 1.0–2.0	0.0–1.0	
mRS improvement ≥ 1 (N)	3	37	0.243	3	15	0.727
%	23.1%	41.1%		27.3%	36.6%	
SH (N)	1	2	0.336	0	1	1.0
%	7.7%	2.2%		0.0%	2.4%	
iHM (N)	2	6	0.265	1	5	1.0
%	15.4%	6.7%		9.1%	12.2%	

Significant values are shown in bold.

*SH* symptomatic intracranial hemorrhage, *iHM* in-house mortality, *SMT* standard medical therapy, *MT* mechanical thrombectomy, *IQR* interquartile range, *mRS* modified rankin scale, *NIHSS* national institute of health stroke scale, *TICI* thrombolysis in cerebral infarction score, *PSM* propensity score matching.

Patients who underwent MT with an angiographic result of below mTICI 2b (within this study setting defined as unsuccessful MT) were compared to the SMT cohort to determine clinical and safety outcomes. No significant difference was found between the groups in NIHSS improvement and mRS improvement (*P* > 0.05). There were no significant differences in the occurrence of symptomatic intracranial hemorrhage or in-house mortality (*P* > 0.2, Table 3b).

## Discussion

In this study, MT was compared with SMT in consecutive acute stroke patients with distal, medium vessel occlusions (DMVO) of the anterior circulation. Most interestingly, patients with successful MT had significantly better clinical improvement and MT did not increase the rate of symptomatic intracranial hemorrhages. This is in line with previous studies on proximal M2 occlusions and occlusions of the posterior cerebral artery^14,15^, as well with initial safety assessments of MT in DMVO^16,17^.

MT has been established as the standard treatment option for patients with acute stroke due to large vessel occlusion (LVO), as primarily demonstrated by five randomized multicenter studies and confirmed by numerous following studies^1,3–5,18^. In contrast, evidence and treatment recommendations for MT in DMVOs remain sparse as RCTs are still missing. However, MT in DMVOs is regularly performed in many comprehensive stroke centers. DMVOs have some unique characteristics that distinguish them from LVOs. First, the clinical presentation of patients with DMVO depends much more on the localization of the perfusion deficit than on its size. As distal occlusions may be better collateralized and show smaller perfusion deficits, clinical scores like the NIHSS are often more favorable in patients with DMVO^19^. However, a low NIHSS can also be associated with life-changing disabilities, especially in patients with aphasia or monoparesis. Second, the use of IV thrombolysis is more effective in distal occlusions. A study with 335 patients showed 44% complete recanalization in distal MCA occlusions compared to 30% in proximal MCA occlusions and 0% in terminal ICA occlusions^20^. Therefore, medical treatment options for DMVO are better than those for LVO, but associated recanalization rates are still far below those of up to 84%, recently reported in dedicated MT in distal occlusions^16^. Additionally, considering the time window and contraindications, IVT is not applicable for all patients (< 38% in our cohort). Third, the recanalization procedure of a DMVO is often more complex because of smaller and more fragile vessels, and thus may lead to more complications such as subarachnoidal hemorrhage compared to LVO^16^.

In summary, mechanical recanalization in DMVO has a different benefit/risk ratio compared to LVO and thus is still controversial, as evidence from direct comparison of MT versus SMT is still lacking. Patients with DMVO in the “big five” RCTs were either excluded or undersampled^21^. Studies analyzing mechanical recanalization of the (mainly proximal) M2 segment of the MCA showed positive results, similar to those observed in M1 occlusions of the MCA^15^. Studies investigating exclusively DMVOs are relatively scarce, and a matched comparison to SMT is lacking so far. Romano et al. described a good clinical benefit and high recanalization rates in 44 patients with distal M2 and M3 occlusions, however, without comparison to SMT^22^. Similar results were recently demonstrated by Fischer et al.^16^.

To the authors’ knowledge, this is the largest study comparing MT to SMT in acute stroke patients with DMVO of the anterior circulation. The mean age of our patients was higher than in the studies described above and the NIHSS at admission was lower (median 7). It was noticeable that patients assigned to MT were more severely affected with a higher NIHSS at admission than patients in the SMT group. This is best explained by the fact that, in the absence of precise guidelines, severely affected patients are more likely to be treated with a more aggressive therapy like MT, as treatment was individually selected by the neurologists and neuroradiologists in charge and was not randomized. However, this bias was eliminated by propensity score matching analysis with the covariates NIHSS and mRS at admission.

To investigate the direct impact of successful recanalization, we compared MT patients with a mTICI ≥ 2b to SMT. This subgroup showed a significantly higher NIHSS improvement and a higher mRS improvement. This can be explained by the high correlation between recanalization rate and clinical outcome known from LVO studies^23^. Similarly, the TOPMOST study investigated MT in DMVO stroke in the posterior circulation and showed favorable results of MT in terms of NIHSS improvement, as in our study^14^.

Patients with acute ischemic stroke due to vascular occlusion but low clinical deficit (NIHSS ≤ 5) are challenging. Alexandre et al. showed in their study that early MT is likely superior to BMT in patients with LVO, even if their NIHSS on admission is low (≤ 5)^24^. However, this is not necessarily the same for DMVO, as another study showed no benefit of early MT compared with BMT in patients with NIHSS ≤ 5 and isolated M2 occlusions^25^. In our study, subgroup analysis could not be performed to address this issue because of the limited number of patients.

The main goal of mechanical thrombectomy is the recanalization of the occluded vessel and the reperfusion of the target territory. The rate of successful recanalization after MT in our patients with DMVO was 72.9% and therefore lower as in selected DMVO studies^14,22,26^. On the other hand, data from the HERMES Collaboration revealed even lower recanalization rates (59.2%) in patients with M2 occlusion undergoing MT^6^. As distal vessels are smaller and tend to be more fragile and tortuous, MT is more complex in DMVO. Interventionalists may discontinue the procedure earlier in fear of complications. Our study was conducted over a long period of time, during which distal recanalization techniques were continuously improved^16,22^. In contrast to the standard approach in LVO, where multiple MT maneuvers are regularly performed, we had several cases (six in total), where MT was aborted unsuccessfully after only one maneuver without an objective reason. Additionally, current stent retrievers and aspiration catheters are mainly designed for LVO, rendering the most effective techniques, like PROTECT PLUS, unfeasible to be performed in DMVO^27^. Haussen et al. investigated different recanalization approaches in DMVO by comparing stent retriever vs. thromboaspiration catheters^26^. The use of stent retriever led to higher rates of reperfusion, but no difference in clinical outcome was shown. Similar results were also obtained by Renieri et al. 2021, however, with lower complication rates with direct aspiration^28^. In our study only one patient was treated by aspiration catheter alone without using a stent retriever, so no conclusion can be reached in this regard. Nevertheless, recanalization techniques in DMVO seem to be particularly important to improve the recanalization rates, which are still below those in LVO. In this context, the rapid development in recent years of small stent retrievers as well as aspiration catheters with larger aspiration flow rates and ingestion forces must be taken into account^29^.

Periprocedural complications were relatively low in our cohort, and symptomatic intracranial hemorrhages were comparable in the MT group and the SMT group, also after matching. Even in the subgroup that included MT without successful reperfusion, the complication rate remained low. This is in line with the studies already mentioned, which also showed a low risk profile^6,14,15,22,26^.

### Limitations

This study is limited by its retrospective and single-center design as well as the lack of long-term follow up data (e.g. mRS after 3 months). Furthermore, we initially observed a strong treatment bias with a higher NIHSS in patients selected for MT. The propensity score matching analysis eradicated this bias but also reduced the statistical power of our analysis (N = 138 vs N = 82 post matching). Thus, we were not able to further subdivide the patient cohort to e.g. investigate distal M2 and M3 occlusions separately.

## Summary

Mechanical thrombectomy in patients with acute ischemic stroke and DMVO appears safe and feasible. This study strengthens the assumption that patients benefit clinically from a successful MT at low complication rates. Although RCTs are still needed for evidence-based treatment recommendations, MT can be considered as a promising and safe treatment option in DMVO.

## Data Availability

The data that support the findings of this study are available on request from the corresponding author, DS.
